# Impaired wound healing is associated with poorer mood and reduced perceived immune fitness during the COVID‐19 pandemic: A retrospective survey

**DOI:** 10.1002/hsr2.764

**Published:** 2022-08-08

**Authors:** Jessica Balikji, Pantea Kiani, Pauline A. Hendriksen, Maarten M. Hoogbergen, Johan Garssen, Joris C. Verster

**Affiliations:** ^1^ Division of Pharmacology, Utrecht Institute for Pharmaceutical Sciences Utrecht University Utrecht The Netherlands; ^2^ Division of Plastic Surgery Catharina Ziekenhuis Eindhoven The Netherlands; ^3^ Global Centre of Excellence Immunology Nutricia Danone Research Utrecht The Netherlands; ^4^ Centre for Human Psychopharmacology Swinburne University Melbourne Victoria Australia

**Keywords:** COVID‐19, mood, perceived immune fitness, slow healing wounds, wound infection

## Abstract

**Background and Aims:**

The coronavirus disease‐2019 (COVID‐19) pandemic disrupted medical care of patients with chronic wounds, and in combination with other negative effects of lockdown measures, this may have a negative effect on mood and quality of life. Until now, the consequences of the COVID‐19 pandemic and associated lockdowns for individuals with impaired wound healing have not been investigated.

**Methods:**

An online survey was conducted to evaluate perceived immune fitness, mood, and health, both before and during the COVID‐19 pandemic.

**Results:**

Of the 331 Dutch pharmacy students that completed the survey, *N *= 42 participants reported slow healing wounds and/or wound infection and were allocated to the impaired wound healing group; the other *N* = 289 participants served as control group. The survey assessed mood, perceived immune fitness, and health correlates for (a) the year 2019 (the period before the lockdown), (b) the first lockdown period (March 15–May 11, 2020), (c) summer 2020 (no lockdown), and (d) the second lockdown (November 2020–April 2021). The analysis revealed that negative mood effects, reductions in quality of life, and perceived immune fitness during the two lockdowns were significantly more pronounced among individuals that reported impaired wound healing compared to the control group. The effects on mood, perceived immune fitness, and health correlates were most pronounced for the second lockdown period.

**Conclusion:**

The COVID‐19 pandemic is associated with significantly poorer mood, quality of life, and reduced perceived immune fitness. These effects are significantly more pronounced among individuals with self‐reported impaired wound healing.

## 1 INTRODUCTION

Due to its rapid worldwide expansion, on March 11, 2020, the World Health Organization declared the 2019 coronavirus disease (COVID‐19) a pandemic.^1^ Lockdown measures to reduce the spread of COVID‐19 (e.g., stay at home orders, closure of schools and businesses) were enforced in many countries, including the Netherlands. A growing body of evidence shows that these lockdown periods had significant negative socioeconomic consequences,^2^, ^3^ and for individuals who have difficulty to cope with lockdown restrictions these periods have been associated with poorer mood (e.g., anxiety, depression, stress, and loneliness) and reduced quality of life.^4^, ^5^

Of particular concern for individuals with chronic diseases and conditions was the impact of the COVID‐19 pandemic on medical care. Due to the high demand of medical services and facilities by patients infected with COVID‐19, delayed care was common practice for less urgent medical interventions and treatments of other patients.^6^, ^7^ However, hospitals and emergency departments also reported a decline in admissions for the treatment of potentially life‐threatening conditions such as myocardial infarction, stroke, or hyperglycemic crisis.^8^, ^9^, ^10^ Also in the Netherlands, the Dutch Healthcare Authority (Nederlandse Zorgauthoriteit) reported a consistent delay in healthcare services across all medical disciplines.^11^ Postponed treatment or delayed diagnosis may also negatively impact long‐term survival.^12^, ^13^ For example, delayed treatment of oncological patients^12^ or disrupted access to rehabilitation care^13^ are likely to have significant negative health consequences, including increased burden of disease or reduced functional outcomes, respectively.^14^

**Figure 1:**
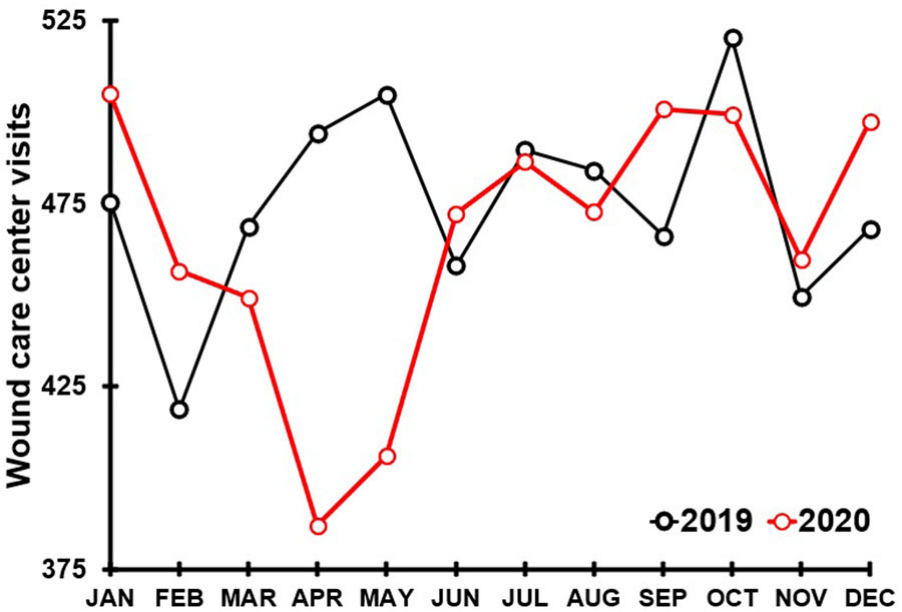
Average number of wound care center visits per month. Note: Aggregate average monthly wound care center visits for 2019 (319 centers) and 2020 (297) centers across the USA. Data provided by Net Health® [30].

Delayed wound care is of particular concern during the COVID‐19 pandemic. Since thesy often have underlying comorbidities such as diabetes, chronic wound patients are at increased risk for worse COVID‐19 outcomes in terms of hospitalization and death. While for some patients a successful transition to online wound management was possible,^15^ more frequently the access to wound care facilities was limited. For example, a survey conducted among Italian medical doctors and nurses^16^ revealed that the COVID‐19 pandemic significantly disrupted the care of patients with chronic wounds. Delayed care is worrisome, given the complications from unmanaged or inadequate wound healing^17^, ^18^, ^19^ such as the increased risk of infection.^20^, ^21^ Particularly diabetic foot ulcer patients need an early referral to reduce the risk of infection, amputation, and subsequent mortality rates.^22^, ^23^, ^24^, ^25^, ^26^ Also, inadequate treatment of vascular leg ulcers is associated with a high risk of lower extremity amputation and increased mortality.^27^ Given that the 5‐year survival of patients with this condition is only 50%–60%,^28^, ^29^ continuous wound care and patient monitoring is critical. In the United States, aggregated data from about 300 wound care centers provided by Net Health® revealed a disruption in hospital outpatient services starting in April 2020 when the first lockdowns were installed. The lockdowns resulted in a decrease in wound care center visits up to 20% compared with pre‐COVID 2019 visits^30^ (see Figure 1).

Interrupted care not only has negative consequences in terms of poor wound management, but it has also been associated with reduced quality of life^31^, ^32^, ^33^, ^34^ and poorer mood and general health.^35^, ^36^, ^37^, ^38^ Chronic wound patients frequently experience multiple issues that relate to a poorer quality of life, including loss of mobility, inability to perform daily activities, and loss of work.^32^, ^34^ Chronic wound patients frequently suffer from wound‐related psychosocial distress, including anxiety and depression,^35^, ^36^, ^37^, ^38^ which can further negatively impact their mental health and quality of life. It is likely that the COVID‐19 lockdown periods and associated delayed care may have an additional negative impact on mood and health of individuals that suffer from impaired wound healing compared to healthy individuals without underlying diseases. Although concerns in this regard have been expressed previously,^39^ up to now, data on the effects of the COVID‐19 pandemic of individuals with impaired wound healing are scarce.^40^, ^41^ Therefore, the current study evaluated to what extent effects of mood and quality of life are more pronounced in individuals with impaired wound healing. The latter is important because this knowledge can be used in future pandemics to minimize the negative effects of preventive measures on patients' well‐being and uninterrupted treatment. To address this gap of knowledge, we examined perceived immune fitness mood and health correlates of the COVID‐19 pandemic in young adults (i.e., pharmacy students, PhD candidates, and postdocs) who reported impaired wound healing and compared the outcomes with those who did not report experiencing impaired wound healing. It was hypothesized that individuals with impaired wound healing report poorer perceived immune fitness, mood, and health correlates, both before and during the COVID‐19 pandemic. In addition, it was hypothesized that the effects of the lockdown periods on these outcomes will be more pronounced in individuals with impaired wound healing compared to the healthy controls.

## 2 MATERIALS AND METHODS

An online retrospective survey was conducted in the first week of June 2021 among students, PhD candidates, and postdocs of the department of pharmaceutical sciences of Utrecht University, The Netherlands. The study was conducted according to the guidelines of the Declaration of Helsinki, and reviewed and approved by the Science‐Geo Ethics Review Board of Utrecht University (protocol code: S‐21525, date of approval: May 19, 2021). All participants gave electronic informed consent. As an incentive participants could enter a prize draw to win one of two Euro 100,‐ vouchers. Participants were invited via email to complete the survey. The survey was designed via SurveyMonkey and took approximately 10 min to complete. Since the department comprises a considerable number of international students, participants could choose to complete the survey in English or Dutch language. A thorough description of the survey content and the data set are published elsewhere.^42^

### 2.1 Immune fitness

To assess immune fitness during the pandemic (the period March 2020–March 2021), the Immune Status Questionnaire (ISQ) was completed.^43^ The ISQ consists of seven items, including “common cold,” “diarrhea,” “sudden high fever,” “headache,” “muscle and joint pain,” “skin problems (e.g., acne and eczema),” and “coughing.” On a 5‐point Likert scale, patients indicated how often participants experienced each item. Answering possibilities comprised “never,” “sometimes,” “regularly,” “often,” and “(al‐most) always.” The sum‐score across the 7 items was computed, and transformed into the final ISQ score ranging from 0 (poor) to 10 (excellent). Two items were added for this study, that is, “slow healing wounds” and ‘wound infection.” If participants indicated that they experienced at least one of these two items during the COVID‐19 pandemic they were allocated to the ‘Impaired wound healing' group. If they did not experience the items they were allocated to the “Control group.”

To assess perceived immune fitness at specific time points, a 1‐item scale ranging from 0 (poor) to 10 (excellent) was used, with higher scores indicating a better perceived immune fitness.^44^ Perceived immune fitness was rated for (a) the year 2019 (the period before the lockdown), (b) the first lockdown period (March 15–May 11, 2020), (c) summer 2020 (no lockdown), and (d) the second lockdown (November 2020–April 2021).

### 2.2 Mood

Mood items included “stress,” “anxiety,” “depression,” “fatigue,” “loneliness,” “optimism,” and “happiness.” All items were scored on a scale ranging from 0 (absent) to 10 (extreme). The use of 1‐item scales has been validated previously.^45^ The items were rated for (a) the year 2019 (the period before COVID‐19), (b) the first lockdown period (March 15–May 11, 2020), (c) summer 2020 (no lockdown), and (d) the second lock‐down (November 2020–April 2021).

### 2.3 Health correlates

Quality of life, and sleep quality were assessed with 1‐item scales from 0 (very poor) to 10 (excellent). Being active was assessed with 1‐item scales from 0 (not at all) to 10 (extremely active). The use of these 1‐item scales has been validated previously.^45^ The items were rated for (a) the year 2019 (the period before COVID‐19), (b) the first lockdown period (March 15–May 11, 2020), (c) summer 2020 (no lockdown), and (d) the second lockdown (November 2020–April 2021).

### 2.4 Statistical analysis

Data were analyzed with SPSS (IBM Corp. Released 2013. IBM SPSS Statistics for Windows, Version 27.0: IBM Corp.). Mean, standard deviation (SD), median, and the interquartile range (IRQ) were computed for all variables, and distributions were checked for normality with the Kolmogorov–Smirnov test and by visual inspection. Since the data were not normally distributed, nonparametric tests were conducted for the statistical analysis.

Between‐group comparisons were conducted with the Independent Samples Mann–Whitney *U* test. A Bonferroni's correct was applied to adjust for multiple comparisons (*p* < 0.0018 for mood outcomes, *p* < 0.0125 for health correlates, *p* < 0.007 for ISQ items, two‐sided). Within‐subject comparisons compared the assessments made for four timepoints (before lockdown, first lockdown, summer, second lockdown). These analyses were conducted with the Related‐Samples Friedman's Two‐Way Analysis of Variance by Ranks test. A Bonferroni's correction was applied, and comparisons were significant if the adjusted *p* value was <0.05 (two‐sided). Finally, Spearman's correlations were computed between all assessed variables for the period before the COVID‐19 pandemic. To account for multiple comparisons, the significance level for correlations with mood outcomes was set at *p* < 0.005 (two‐sided).

## 3 RESULTS

**Figure 2:**
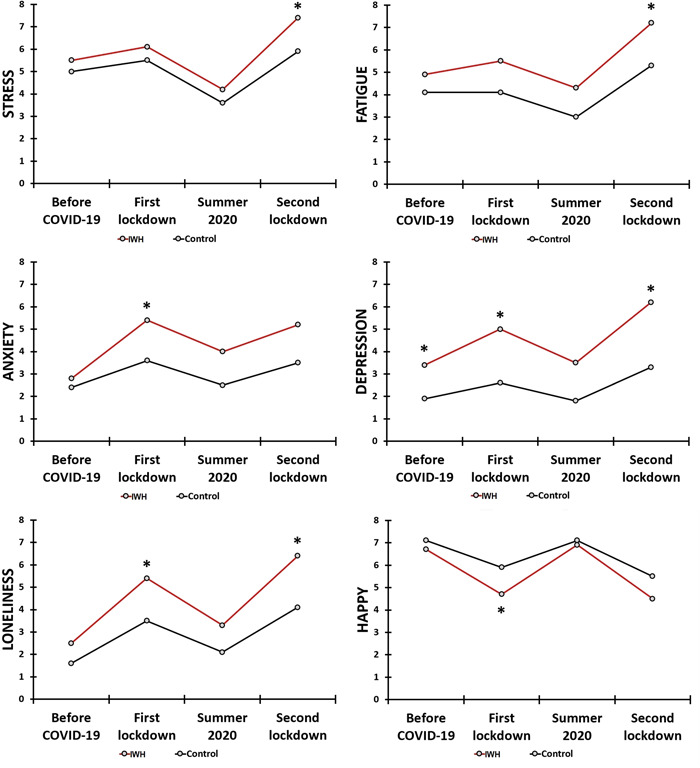
Mood during the COVID‐19 pandemic. Note: Assessments are shown for the ‘Impaired wound healing (IWH) group and control group. Significant differences between the groups (*p* < 0.0018, after Bonferroni's correction for multiple comparisons) are indicated by *. COVID‐19, coronavirus disease‐2019

*N* = 341 participants completed the survey. Of these, 10 participants did not report on their wound healing status and were excluded from the analysis, The mean (SD) of the sample (*N* = 331) was 23.0 (4.2) years old and 74.9% of the participants (248/331) were women. Forty‐two participants were allocated to the “Impaired Wound Healing” (IWH) group, and 289 participants to the “Control” group. Table 1 and Figure 2 summarize the mood assessments for the IWH and control group.

**Table 1:** Mood before and during COVID‐19

Mood		Before COVID‐19		First lockdown		Summer 2020		Second lockdown	
Variable		IWH	Control	IWH	Control	IWH	Control	IWH	Control
Stress	Mean (SD)	5.5 (2.1)	5.0 (2.2)	6.1 (2.6)	5.5 (2.6)^a^	4.2 (2.6)^b^	3.6 (2.5)^a,b^	7.4 (2.0)^a,c^	5.9 (2.5)*,^a,c^
	Median (IQR)	6.0 (3.0)	5.0 (3.0)	7.0 (4.0)	6.0 (4.25)	4.0 (4.0)	3.0 (4.0)	8.0 (3.0)	6.0 (4.0)
Anxiety	Mean (SD)	2.8 (2.8)	2.4 (2.6)	5.4 (2.8)^a^	3.6 (2.9)*,^a^	4.0 (2.7)^b^	2.5 (2.5)^b^	5.2 (3.1)^a^	3.5 (3.1)^a,c^
	Median (IQR)	2.0 (5.0)	2.0 (4.0)	6.0 (3.0)	3.0 (5.0)	4.0 (4.0)	2.0 (4.0)	6.0 (4.0)	3.0 (6.0)
Depression	Mean (SD)	3.4 (2.8)	1.9 (2.5)*	5.0 (3.0)	2.6 (2.8)*,^a^	3.5 (3.1)	1.8 (2.3)^b^	6.2 (2.8)^a,c^	3.3 (3.0)*,^a,b,c^
	Median (IQR)	3.0 (4.0)	1.0 (3.0)	5.0 (6.0)	2.0 (4.0)	4.0 (6.0)	1.0 (3.0)	7.0 (4.0)	3.0 (6.0)
Fatigue	Mean (SD)	4.9 (2.5)	4.1 (2.6)	5.5 (3.2)	4.1 (2.6)	4.3 (3.0)	3.0 (2.5)^a,b^	7.2 (2.3)^a,b,c^	5.3 (2.7)*,^a,b,c^
	Median (IQR)	4.0 (4.0)	4.0 (4.0)	5.0 (6.0)	4.0 (4.0)	5.0 (5.0)	3.0 (4.0)	8.0 (4.0)	5.0 (4.25)
Loneliness	Mean (SD)	2.5 (2.2)	1.6 (2.0)	5.4 (2.8)^a^	3.5 (2.9)*,^a^	3.3 (2.5)	2.1 (2.4)^b^	6.4 (2.9)^a,c^	4.1 (3.1)*,^a,c^
	Median (IQR)	2.0 (4.0)	1.0 (3.0)	5.0 (4.0)	3.0 (5.0)	3.0 (4.0)	1.0 (4.0)	7.0 (3.0)	4.0 (6.0)
Optimism	Mean (SD)	6.9 (1.5)	7.1 (2.0)	4.7 (2.2)^a^	6.0 (2.0)^a^	6.3 (1.7)^b^	6.8 (1.9)^b^	5.1 (2.3)^a,c^	5.4 (2.1)^a,b,c^
	Median (IQR)	7.0 (2.0)	8.0 (2.0)	4.0 (4.0)	6.0 (2.0)	6.0 (3.0)	7.0 (2.0)	5.0 (4.0)	6.0 (3.0)
Happiness	Mean (SD)	6.7 (1.9)	7.1 (1.8)	4.7 (1.8)^a^	5.9 (2.0)*,^a^	6.9 (1.4)^b^	7.1 (1.8)^b^	4.5 (2.2)^a,c^	5.5 (1.9)^a,b,c^
	Median (IQR)	7.0 (3.0)	7.0 (2.0)	5.0 (2.0)	6.0 (2.0)	7.0 (2.0)	7.0 (2.0)	5.0 (3.0)	6.0 (3.0)

*Note*: Mean, standard deviation, median, and interquartile range are shown. Significant differences (*p* < 0.0018, applying a Bonferroni's correction for multiple comparisons) between the IWH group and control group are indicated by *. Significant within‐subject differences (adjusted *p*‐values < 0.05, applying a Bonferroni's correction for multiple comparisons) are indicated as follows: a = significantly different from “before COVID‐19”, b = significant difference from the “first lockdown”, c = significant difference from “summer 2020”.

Abbreviations: COVID‐19, coronavirus disease‐2019; IQR, interquartile range; IWH, impaired wound healing; SD, standard deviation.

Differences in mood ratings between the groups before the COVID‐19 pandemic were only statistically significant for depression, showing significant higher ratings for the IWH group. The analysis revealed that reduced mood was evident during the COVID‐19 pandemic, both among individuals with impaired wound healing as well as the control group. The effects were greatest throughout the two lockdown periods. As is evident from Table 1 and Figure 2, the negative mood effects during the two lockdown periods were significantly more pronounced among individuals who reported impaired wound healing compared to the control group.

Table 2 summarizes the assessments of health correlates. No significant differences between the groups were present before the COVID‐19 pandemic. For both groups, sleep quality, being active, and quality of life were significantly reduced during the COVID‐19 pandemic. Significant differences between the groups were found for the two lockdown periods. The impaired wound healing group reported significantly greater reductions in sleep quality (second lockdown period) and quality of life (both first and second lockdown) compared to the reductions among the control group. The assessment of perceived immune fitness revealed a significant reduction in the impaired wound healing group during the first and second lockdown period, as well as the lockdown‐free summer period (see Table 2). No significant differences were found between the groups for being active.

**Table 2:** Health correlates during COVID‐19

Mood		Before COVID‐19		First lockdown		Summer 2020		Second lockdown	
Variable		IWH	Control	IWH	Control	IWH	Control	IWH	Control
Being active	Mean (SD)	6.0 (2.8)	6.2 (2.6)	3.7 (2.9)^a^	4.8 (2.9)^a^	4.5 (2.8)	5.5 (2.6)^a,b^	3.4 (2.7)^a^	4.6 (2.8)^a,c^
	Median (IQR)	6.0 (4.0)	7.0 (3.0)	3.0 (4.0)	5.0 (5.0)	4.0 (4.0)	6.0 (4.0)	3.0 (4.0)	5.0 (5.0)
Quality of life	Mean (SD)	7.3 (1.5)	7.7 (1.3)	5.1 (2.2)^a^	6.4 (1.8)*,^a^	7.1 (1.5)^b^	7.4 (1.5)^b^	4.7 (2.1)^a,c^	6.0 (1.9)*,^a,c^
	Median (IQR)	8.0 (2.0)	8.0 (1.0)	6.0 (4.0)	7.0 (3.0)	7.0 (2.0)	8.0 (1.0)	5.0 (3.0)	6.0 (2.0)
Sleep quality	Mean (SD)	6.9 (1.8)	7.0 (1.8)	6.5 (2.2)	6.8 (2.0)^a^	6.7 (2.0)	7.2 (1.7)^b^	5.1 (2.0)^a,c^	6.4 (2.1)*,^a,b,c^
	Median (IQR)	7.0 (2.0)	7.0 (2.0)	7.0 (3.0)	7.0 (2.0)	7.0 (3.0)	7.0 (2.0)	5.0 (3.0)	7.0 (3.0)
Perceived immune functioning	Mean (SD)	7.1 (1.7)	7.6 (1.7)	6.2 (2.1)	7.3 (1.9)*	6.6 (1.8)	7.5 (1.7)*	5.6 (2.2)^a^	7.1 (1.8)*,^a,c^
	Median (IQR)	8.0 (1.0)	8.0 (2.0)	6.0 (3.0)	7.0 (2.0)	7.0 (3.0)	8.0 (1.0)	6.0 (2.0)	7.0 (2.0)

Note: Mean, standard deviation, median, and interquartile range are shown. Significant differences (*p* < 0.0125, applying a Bonferroni's correction for multiple comparisons) between the IWH group and control group are indicated by *. Significant within‐subject differences (adjusted *p*‐values < 0.05, applying a Bonferroni's correction for multiple comparisons) are indicated as follows: a = significantly different from “before COVID‐19”, b = significantly different from the “first lockdown”, c = significantly different from “summer 2020”.

Abbreviations: COVID‐19, coronavirus disease‐2019; IQR, interquartile range; IWH, impaired wound healing; SD, standard deviation.

Table 3 shows immune status during the COVID‐19 pandemic as assessed with the ISQ. The analysis of ISQ data revealed that the single‐item perceived immune fitness ratings were significantly poorer in the impaired wound healing group than the control group. In particular, the impaired wound healing group reported significantly higher frequencies of experiencing diarrhea, headache, muscle and joint pain, skin problems, and coughing.

**Table 3:** Immune status during the COVID‐19 pandemic

ISQ		During COVID‐19		
Items		IWH	Control	*p* Value
Common cold	Mean (SD)	0.9 (0.8)	0.7 (0.8)	0.138
	Median (IQR)	1.0 (4.0)	1.0 (1.0)	
Diarrhea	Mean (SD)	1.1 (0.9)	0.6 (0.7)	<0.001*
	Median (IQR)	1.0 (1.25)	0.0 (1.0)	
Sudden high fever	Mean (SD)	0.2 (0.4)	0.1 (0.3)	0.040
	Median (IQR)	0.0 (0.0)	0.0 (0.0)	
Headache	Mean (SD)	2.0 (1.1)	1.3 (0.9)	<0.001*
	Median (IQR)	2.0 (2.0)	1.0 (1.0)	
Muscle and joint pain	Mean (SD)	1.1 (0.8)	0.7 (0.8)	0.001*
	Median (IQR)	1.0 (1.0)	1.0 (1.0)	
Skin problems (e.g., acne and eczema)	Mean (SD)	1.5 (1.2)	0.9 (1.1)	0.003*
	Median (IQR)	1.0 (2.25)	1.0 (2.0)	
Coughing	Mean (SD)	1.0 (0.8)	0.6 (0.7)	<0.001*
	Median (IQR)	1.0 (0.25)	0.0 (1.0)	
ISQ	Mean (SD)	5.7 (2.4)	7.7 (2.1)	<0.001*
	Median (IQR)	6.0 (3.25)	8.0 (3.0)	

*Note*: Mean, standard deviation, median, and interquartile range are shown. Significant differences between the IWH group and control group (*p*‐value < 0.007, after Bonferroni's correction for multiple comparisons) are indicated by *.

Abbreviations: COVID‐19, coronavirus disease‐2019; IQR, interquartile range; IWH, impaired wound healing; SD, standard deviation.

**Figure 3:**
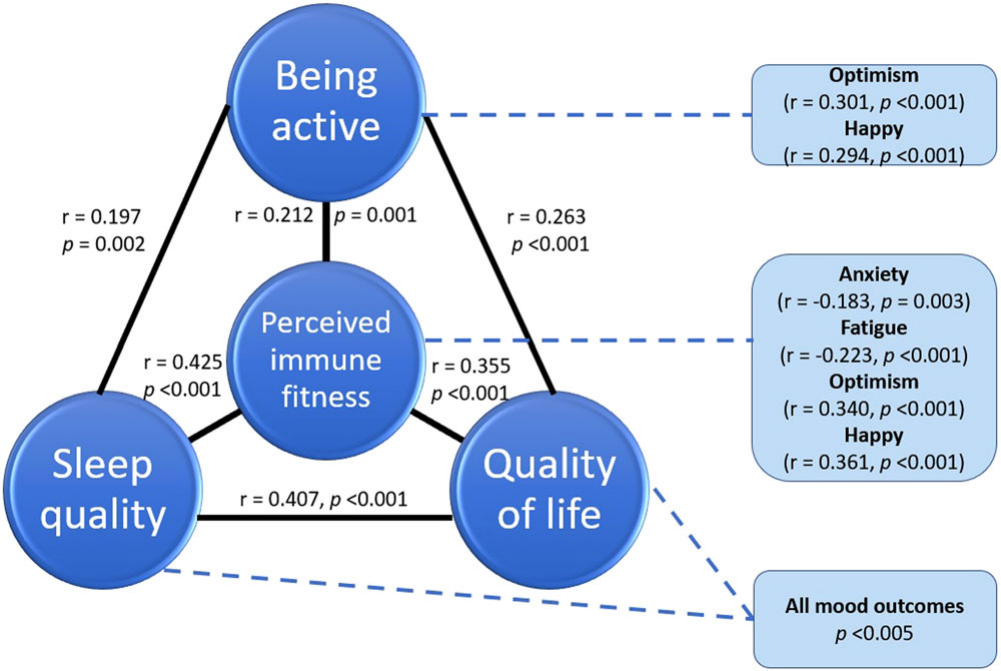
Relationship of the assessed mood and health correlates with perceived immune fitness. *Note*: Only significant correlations (*p* < 0.005, after Bonferroni's correction) are shown.

Finally, it must be noted that the variables assessed in this study strongly correlate with each other. This is illustrated in Figure 3, which shows Spearman's correlations between the variables for the pre‐COVID‐19 period. Only significant correlations are shown (*p* < 0.005, after Bonferroni's correction). The correlations between the individual mood variables, which were all significant (*p* < 0.001), have been omitted from Figure 3.

## 4 DISCUSSION

The current study confirms that poorer immune functioning during the COVID‐19 pandemic is characteristic for individuals that reported impaired wound healing. Individuals with impaired wound healing also reported significantly greater negative mood effects and poorer quality of life compared to the control group, which were most pronounced during the second lockdown period. These findings may be explained by pre‐COVID literature, which reports that patients with impaired wound healing suffer from wound‐related psychosocial distress, such as increased anxiety and depression.^35^, ^36^, ^37^, ^38^ Recent studies found that individuals with self‐reported impaired wound healing also reported significantly poorer mood,^46^ significantly more gastrointestinal complaints,^47^ and significantly poorer immune fitness^48^ that the control group (reporting normal wound healing).

The effects of the lockdown periods were significantly more pronounced in individuals with self‐reported impaired wound healing. It may be hypothesized that interrupted or poor wound management due to delayed care during the pandemic may have further worsened immune fitness, mood, and quality of life of individuals with impaired wound healing. However, as no information on (delayed) treatment was collected in the current study, this should be verified in future studies, preferably in formally diagnosed and monitored patients.

In the current study, pre‐COVID‐19 data show that the health correlates are highly associated with perceived immune fitness, as well as with each other (See Figure 3). This observation suggests that improving one of these variables (e.g., sleep quality) will have a direct effect on perceived immune fitness and mood of patients with impaired wound healing. Although tempting, however, causational conclusions cannot be drawn based on the current correlational analysis. To establish the nature of these interactions, additional prospective intervention studies are needed. To our knowledge, this is the first study evaluating mood and immune fitness during the COVID‐19 pandemic in relation to impaired wound healing. Furthermore, pre‐COVID literature on mood and quality of life of patients with chronic wounds is scarce. This is unfortunate, as studies in other areas of medicine have demonstrated that positive mood and mental resilience contribute to both treatment compliance and recovery.^49^, ^50^ Given this, more research on the well‐being of chronic wound patients is needed.

In the current study, across both groups, the COVID‐19 lockdown periods were associated with negative mood, reduced quality of life, and poorer immune fitness. This has also been reported in several other studies that evaluated these parameters in the general population, using a comparable survey methodology and used the same assessment scales.^51^, ^52^ Thus, the current findings are in line with previous research. The study adds to the literature that these effects are significantly more pronounced in individuals with self‐reported impaired wound healing.

To interpret the data correctly, several limitations of the study should be considered. First, the data were self‐reported and collected retrospectively. As such, recall bias may have influenced the study outcomes. Prospective studies with real‐time assessments should confirm our findings. Second, participants were allocated to the impaired wound healing group or control group. It is important to note that this study was based on self‐reported data and no formal diagnosis was obtained to verify this. Third, the sample size of the current study was relatively small and comprised a convenience sample of Dutch students. Therefore, it is unclear to what extent our findings can be generalized to other age groups or extrapolated to the general population. Also, in line with the sex distribution at Utrecht University, females were overrepresented in the sample. However, the sample size was too small to evaluate possible sex differences. It is also unclear to what extent the findings in this nonclinical sample can be translated to mood and quality of life of diagnosed patients with impaired wound healing, such as patients with diabetic foot ulcer. In the current study it was not assessed whether participants had diabetes. However, the fact that our findings on mood and quality of life are already present in a nonclinical population strengthen our observation that these effects will also be present in formally diagnosed patients. Finally, the presented correlations do not imply causality, and directional conclusions cannot be drawn from the data.

To conclude, compared to pre‐COVID, during the COVID‐19 pandemic significant reductions in mood, perceived immune functioning and health correlates were reported for both groups. However, individuals that reported impaired wound healing during the COVID‐19 pandemic reported significantly poorer mood and a significantly greater reduction in perceived immune fitness when compared to the control group. In general, associations between impaired wound healing, mood, and perceived immune fitness, including factors that would potentially improve wound healing, deserve more research attention.

## TRANSPARENCY STATEMENT

Joris Cornelis Verster affirms that this manuscript is an honest, accurate, and transparent account of the study being reported; that no important aspects of the study have been omitted; and that any discrepancies from the study as planned (and, if relevant, registered) have been explained.

## AUTHOR CONTRIBUTIONS

**Jessica Balikji**: Conceptualization; writing—original draft. **Pantea Kiani**: Conceptualization; methodology; writing—review & editing. **Pauline Anne Hendriksen**: Conceptualization; investigation; methodology; writing—review & editing. **Maarten Michael Hoogbergen**: Conceptualization; writing—review & editing. **Johan Garssen**: Conceptualization; writing—review & editing. **Joris Cornelis Verster**: Conceptualization; data curation; formal analysis; writing—original draft; writing—review & editing.

## CONFLICTS OF INTEREST

Over the past 3 years, J.C.V. has acted as a consultant/advisor for KNMP, Mentis, Red Bull, Sen‐Jam Pharmaceutical, and Toast!. J.G. is part‐time employee of Nutricia Research and received research grants from Nutricia research foundation, Top Institute Pharma, Top Institute Food and Nutrition, GSK, STW, NWO, Friesland Campina, CCC, Raak‐Pro, and EU. The other authors have no potential conflicts of interest to disclose.

## INSTITUTIONAL REVIEW BOARD STATEMENT

The study was conducted according to the guidelines of the Declaration of Helsinki, and approved by the Science‐Geo Ethics Review Board of Utrecht University (protocol code: S‐21525, date of approval: May 19, 2021).

## INFORMED CONSENT STATEMENT

Informed consent was obtained from all participants that took part in the study.

## Data Availability

The data is published open access in the journal MDPI Data and available online at doi:10.3390/data6110120.
